# Trends in the Utilization of Human Papillomavirus Vaccines and the Incidence of Malignant Cervical Cancer in Women and Teenagers: A Secondary Analysis

**DOI:** 10.3390/healthcare10071211

**Published:** 2022-06-28

**Authors:** Myah Luna, Soumya Upadhyay

**Affiliations:** 1 IRB Research Compliance Administrator, University of South Florida, Tampa, FL 33612, USA; lunam1@usf.edu; 2 Healthcare Administration and Policy, School of Public Health, University of Nevada Las Vegas, Las Vegas, NV 89119, USA

**Keywords:** cervical cancer, HPV, vaccinations, adolescents

## Abstract

Background: Human papillomavirus (HPV) is a sexually transmitted infection, and HPV types 16 and 18 are responsible for approximately 66% of all U.S. cervical cancer cases in women. The quadrivalent HPV vaccine was licensed in mid-2006, and it was designed to target and protect against HPV types 6, 11, 16, and 18. The aim of this study is to examine the utilization rate of the HPV vaccine, and the trends and incidence rate of malignant cervical cancer across the United States. Methods: This study utilized data from Surveillance, Epidemiology, and End Results (SEER) and the National Immunization Survey’s (NIS) teenage datasets across select years. For the SEER survey, the modification for confidence intervals by Tiwari et al., 2006, was utilized to obtain the incidence rate per 100,000, so that it could be age-adjusted for the 2000 U.S. standard population, as noted in the data provided by the U.S. Census. The dates examined started in the year 2000 and ended in 2017. For the NIS-Teen survey, the public-use data file was used, and a point estimate (%), with a 95% confidence interval, was performed to examine the trends in HPV vaccine utilization across the U.S. adolescent female population from the years 2007 to 2019. Results: This study found that the rate of diagnosis had been falling over the nearly two decades examined in this study. Implications: This study would support current efforts to encourage the utilization of HPV vaccines that are currently in the vaccination schedule rotation, and to illustrate the importance of completing all doses of the three-step series.

## 1. Introduction

The second most common tumor found in women worldwide is cervical cancer, and the sexually transmitted infection, human papillomavirus (HPV) is the cause of more than 80% of all cervical cancers. It is estimated that currently 79 million Americans are infected with a type of HPV [1]. HPV is an infection that is either benign, or it can cause several cancers in addition to just cervical cancer, depending on the type of HPV. Its high rate of prevalence is both startling and dangerous to the health of millions of Americans. It is estimated that in 2020, about 13,800 new incidences of invasive cervical cancer will be detected, and in this year alone, 4290 women will die as a result of cervical cancer [2] (pp. 321–346).

It has been revealed that in the U.S., cervical cancer disproportionally affects some racial/ethnic groups at higher rates than White women. Hispanic women were found to have a 9.2 rate of new cases per 100,000, compared with a rate of 7.2 in White women. Black women were also detected as having higher rates of new cases, with a score of 8.7, and Native American/Alaskan Native women had the next highest score with a rate of 7.9. The most frequent age of cervical cancer diagnosis is 35–44, but the median age of diagnosis is 50 based on national cancer registry data [3]. The five-year survival rate for cervical cancer is 66.1%, meaning that 33.9% of those diagnosed with cervical cancer die within five years of diagnosis [3]. The reasoning for these disproportionate rates of diagnosis have not yet been solidly determined, but it is likely that the disproportionate distributions of education and wealth play a key role in this phenomenon.

HPV is the most common sexually transmitted infection, and it is estimated that in the United States, those who have had just one sexual partner have at least an 84% lifetime probability of becoming infected [4] (pp. 660–664). A prospective study on college women found that 24 months after the first time these women had intercourse, 40% of the participants had a type of HPV, and 10.8% of those cases had HPV type 16, a known causal agent in cervical cancer development [5].

The detection of HPV in the tissue of cervical cancer biopsies was discovered by German virologist, Harald zur Hausen, and his team in late 1979. With continued examination, they were able to isolate HPV-16 in 1983, and HPV-18 in 1984, from within the DNA of cervical cancer cells [6] (pp. 342–350). This discovery triggered a worldwide effort among scientists and clinicians to create prophylactic vaccines to fight against HPV; these endeavors resulted in a discovery that has the ability to prevent 70–80% of cervical cancer cases globally [7] (pp. 889–899). A meta-analysis performed in 2007 estimates that about 291 million women had HPV DNA at a given time, and about 23% of those infections resulted in HPV-16 and 8.5% resulted in HPV-18 {7}. In mid-2006, the quadrivalent HPV vaccine was licensed for females, and it targeted HPV types 6, 11, 16, and 18. At the time, it was estimated that 6.2 million people were newly infected with HPV each year [8]. By late 2009, the male vaccine was also licensed, but it was bivalent, targeting only HPV types 6 and 11, since its design focus was largely on the prevention of genital warts caused by HPV infections [9] (pp. 630–632). These are concerning estimates, as in the United States alone, HPV-16 and 18 are believed to be responsible for 66% of cervical cancer cases [5].

Since this discovery, other variations of the vaccine have been licensed, targeting even more HPV types, though types 16 and 18 continue to be considered the most threatening. The utilization of these vaccines has been promoted and introduced into the recommended vaccine routine for females, beginning at age 11–12, with catch-up dose vaccinations approved to age 26 to enable the completion of the three-dose series.

Since 2006, over 100 countries have licensed HPV vaccines, and the United States was one of the first countries to introduce the vaccines into its population [10] (p. 1225). Starting in 2012, at least 40 countries added HPV vaccines to their national immunization programs. This acceptance of the HPV vaccine is positive for the health of the global population, but although vaccines are supported by these countries, for those who started the vaccination series, the success rate in terms of how many people complete the injection series is not yet at 100%. Many begin the vaccination series but fail to obtain all three doses, meaning that they may not be effectively protected against dangerous strains of HPV. It is strongly recommended that people complete the series, even if they have interrupted the designated dose schedule, to increase the possible effectiveness of the vaccine [11]. The objective of this study is twofold: (1) examine the trends and incidence rate of malignant cervical cancer across the United States population and (2) to examine the utilization rates of the human papillomavirus vaccine in the adolescent female population of the United States. The findings of this study can inform researchers for future investigations, and give healthcare and public health professionals information to perhaps improve future policy and procedure.

## 2. Literature Review

The utilization of the human papillomavirus vaccine, since it first gained its license, has steadily increased, according to studies performed by other researchers. Despite this, we still have not yet been able to meet the HPV Cancer Free vaccination rate goals set by the American Cancer Society. Indeed, the target currently aims for an 80% vaccination initiation rate across the 13-year-old female population in the United States by the year 2026 [2] (pp. 321–346). To illustrate some of the progression we have been able to detect since the vaccine was licensed, we can first look at the study performed by Dr. Kester and their team in 2012. In this study, they examined a sample of 501 mother–daughter pairs, and they were able to determine from their sample that 51.1% of the participants had intentions of initiating a HPV vaccination, but only 38.8% of them had already completed the vaccination series at the time of interview [12] (pp. 879–885). Furthermore, a study performed by the researcher, Tanja Y. Walker, and their colleagues utilized a national survey and found that 68.1% of adolescents aged 13 to 17 had received at least the first dose of the HPV vaccination series [13] (p. 1109). Sadly, they also found that only 51.1% were up to date with their vaccination schedule. Though these results illustrate that we are possibly seeing an increase in terms of initiation, we also note that the risks for patients who do not complete their vaccination series are still present. This is concerning, because staying on schedule with the vaccination series is very important when it comes to the effectiveness of the protection offered by the vaccine.

When examining the distribution of the utilization of the vaccines across ethnicities in the population, mixed results have been found. Some studies have shown decreased rates of vaccine series completion in non-White populations across the United States, whereas others have found no evidence of this. Dr. Andrea N. Polonijo and Dr. Richard M. Carpiano analyzed National Immunization Survey (NIS) data pertaining to teenagers from 2008 to 2010, and in their sample pool of the population they found that Black adolescents were 22% less likely to initiate the HPV vaccination series compared with White adolescents (2013). The authors have further argued that populations that are more privileged in terms of wealth, knowledge, education, money, and status, are able to benefit from health promoting resources compared with people who do not have these advantages. Dr. Madina Agénor and their colleagues examined the 2015 National Health Interview Survey (NHIS) in search of racial/ethnic disparities in the initiation and completion of the HPV vaccination series across the female population. In their results, they found that initiating vaccination did not have statistically significant differentiations across race/ethnicity, but they did find a differentiation across race/ethnicity when focusing upon the completion of the vaccination series [14] (pp. 393–407). More specifically, in comparison to White women, Black, Latina, and Asian women were found to have significantly lower odds of completing the three-dose HPV vaccination series. This further supports the idea that parent or guardian knowledge about the HPV vaccine, health professional recommendations to receive the HPV vaccine, and the uptake of HPV vaccine, including the initial and follow-up dosages, are important considerations when determining racial inequities regarding the HPV vaccine.

When examining the differentiation of initiation and completion across racial/ethnic groups, researchers also offered possible explanations for these unequal distributions. The most common similarity between all of their proposed explanations was access to information on the human papillomavirus and its dangers, the safety of the HPV vaccine, and the effectiveness of the HPV vaccine in protecting against dangers associated with HPV infection. Plonijo and Carpiano offered the most in-depth analysis, and they identified several factors that likely contributed to the reduced rate of initiation and completion of vaccination in Black adolescents in the U.S. population. In their research, they identified that those with families of lower socioeconomic status had statistically lower odds of possessing knowledge of the HPV vaccine [15] (pp. 115–125). They found that the odds of mothers knowing about the HPV vaccine were 73% lower in mothers who did not possess a high school diploma than in mothers who possessed at least a bachelor’s degree. With regard to mothers who had completed high school and possessed a diploma, they still found that these mothers were 58% less likely to know about the HPV vaccine when compared with bachelor’s degree holding mothers. Moreover, at the time when this research was conducted, the researchers found that those who identified as having a higher-income status were the most educated about the existence of the vaccine, whereas those of lower- and middle-income status were far less likely to possess this knowledge. In the U.S., Black Americans have the lowest average household pretax income, followed closely by Hispanics, which illustrates how the disproportionate distribution of wealth often positions Black Americans in a lower SES position [16]. This information might explain at least part of the reason why Black members of the U.S. population are less likely to initiate and complete the HPV vaccination series. Despite this, the researchers also stated that more investigation into cultural differences is necessary to determine if cultural views play a role in the initiation and completion of the vaccination series for the non-White populations that display reduced completion rates.

Another important factor identified by the discussed researchers is communication with healthcare professionals. The recommendation of HPV vaccine utilization provided by physicians and medical professionals proved to have a measurable influence on the rate of initiation and completion across nearly every study discussed in this literature review. The study performed by Dr. Kester and their colleagues examined qualitative data, and their results found that about 90% of those in their sample, who had successfully initiated HPV vaccinations, reported discussing the vaccine with their provider prior to vaccination, and 88% of them reported that their provider “strongly recommended” vaccination [12] (pp. 879–885). Although this demonstrates how positive recommendations given by providers can influence patients’ decisions to vaccinate, studies have shown the extent to which this area has been neglected since the HPV vaccine was first licensed. A study performed in 2009, three years after the vaccine was licensed, looked to examine the frequency with which physicians recommended the HPV vaccine, and of the physicians surveyed, they found that only about 35% answered that they “always” recommend the HPV vaccine to patients aged 11–12 [17] (pp. 8634–8641). Since 2009, we have seen a large increase in physician support for the HPV vaccine. In 2017, a similar study was conducted, and it revealed that 79% of the physicians who participated in the survey answered that they “always” or “usually” recommend the vaccine to eligible female patients [18] (p. 6122). This increase is hugely encouraging, since we already know how valuable physician recommendations are for patient decision-making processes. The American Medical Association has taken the position of being very supportive of the utilization of the HPV vaccine, publishing several articles discussing the success rate of HPV prevention, and it even offers suggestions as to how to best communicate with patients to encourage vaccination [19]. With the scientific data and the support of respected scientific associations that are vocal within the medical community, the hope is that physicians and healthcare professionals will continue to recommend HPV vaccine utilization in the future. Physician support and recommendations made to patients will be hugely valuable if we want to continue to increase utilization rates across the population.

The objectives of this study are to (1) examine the trends and incidence rate of malignant cervical cancer across the United States population and (2) to examine the utilization rates of the human papillomavirus vaccine in the adolescent female population of the United States.

## 3. Methods

### 3.1. Ethics and Consent

The data collected and included in the Surveillance, Epidemiology, and End Results (SEER) dataset are collected through state registries, and the consent practices are different according to each state’s individual regulations. All data included in the SEER dataset are non-identifiable, thus protecting participant identities. The National Immunization Survey (NIS) keeps all respondents’ personal information concealed, as per federal law through the Public Health Service Act. All CDC staff and contractor staff signed NIS-Teen confidentiality agreements prior to handling data. All participants of the NIS must give informed consent prior to their phone questionnaire, and any health records shared are only acquired if the participant orally consents. Next, each provider was mailed an immunization history questionnaire (IHQ). The University of Nevada, Las Vegas Internal Review Board, stated that since the data used in this study was shared for public-use, and was performed without the 18 HIPAA identifiers, it does not qualify as having used a “human subject”, and therefore, it did not need to be submitted for approval.

### 3.2. Study Design

This study consists of two parts. Part one is an incidence rate analysis of malignant cervical cancer cases in the United States during the years 2000 to 2017. The second part is a trend analysis of HPV vaccine utilization estimates from the year 2007 to 2019 in the adolescent female population of the United States. This study utilized dates from the Surveillance, Epidemiology, and End Results (SEER) and the National Immunization Survey’s (NIS) public-use datasets that concern teenagers.

### 3.3. Analysis

The SEER data was analyzed using the required SEERStat system, which is controlled through the National Cancer Institute, a part of the National Institute of Health. Within the SEERStat system, the SEER Research Data 18 Registries dataset was used, and incidence trend analysis was carried out within the system. For the SEER survey, the modification of confidence intervals by Tiwari et al., 2006, was utilized to obtain the incidence rate per 100,000, which was age-adjusted for the 2000 U.S. standard population, as described in the data provided by the U.S. Census. The dates examined start in the year 2000 and end in 2017.

For the NIS-Teen survey, the data was analyzed using SAS, and the results describe the HPV vaccination female coverage estimates measured by the sample. The estimates are presented as a point estimate (%), with a 95% confidence interval to examine the trends in HPV vaccine utilization across the adolescent female population during the years 2007 to 2019. The data is adjusted for those whose parents did not participate, those living in households without telephones, and those whose vaccination records were not shared by their provider.

### 3.4. Study Population

Inclusion criteria of this study were limited to those who were assigned female at birth in both datasets; this is because both datasets have their own extensive exclusion criteria which did not illustrate the need for further additions to be made. Excluded from this study will be those who did not meet the inclusion criteria set out by the datasets being analyzed. SEER exclusion includes those who are/have: male genitalia, non-malignant tumors, any tumor site outside of the “Cervix Uteri” classification, and those under the age of 19 at time of diagnosis. NIS-Teen exclusion includes those who are/have: outside the ages of 13–17, incomplete screening of the household, and those with incomplete household surveys.

SEER: Data provided is routinely collected on an annual basis, and the data includes patient demographics, the primary tumor site, tumor morphology and stage at diagnosis, the first course of treatment, and follow-ups for vital status. All mortality information published by SEER is provided by the National Center for Health Statistics. The staff of the National Cancer Institute, the organization that runs the SEER data collection, work closely with the North American Association of Central Cancer Registries (NAACCR) to guide state registries with their archiving systems to improve compatibility for the pooling of data. The data represents approximately 34.6% of the U.S. population, all collected from population-based cancer registries. The race/ethnicity of the participants are 31.4% White, 30% of African American, 44% Hispanic, 49.3% Native American, 57.5% Asian, and 68.5% Hawaiian/Pacific Islander.

NIS-Teen: Surveys are conducted of those who are deemed eligible based on the age of the adolescent members in a household. One member of the household must be at least 19–35 months of age for the NIS-Child survey, and the NIS-Teen survey is an extension of the NIS-Child survey. All eligible household adolescents must be rostered, then, one eligible member will be selected randomly, and the questionnaire will only be administered to that one selected eligible member per household. It collects data from 56 geographic areas across the country, states, select cities, and territories. It is a national survey that targets adolescents aged 13 to 17, and to achieve a coefficient of variation (CV), they need to have adequate provider data, which equates to 230 eligible adolescents per estimation area. The questionnaire is designed around the vaccination schedule dictated in the ‘Recommended Immunization Schedule for Children and Adolescents Aged 18 Years or Younger’, which is updated annually as new vaccines are added by the Advisory Committee on Immunization Practices. After being vetted to ensure the eligibility criteria were met, in 2017, the landline sample resulted in 6678 household interviews, and the cell phone sample resulted in 37,007 eligible household interviews. The surveys collected data on vaccination history, the teenager’s health and household health, demographic and socioeconomic information, and the Health Insurance Module (HIM). The sample of data released in the public-use file contains over 20,000 participants from the nationwide survey.

### 3.5. Limitations

Due to the fact that we do not have longitudinal data, a correlation analysis could not be performed with the data that are currently available. Given that the median age of cervical cancer diagnosis is 50, it is not yet possible to determine if those vaccinated in the first year that the vaccine was licensed are connected to any reduction in the malignant cervical cancer diagnoses that are currently being seen across the population. In addition, both datasets have extensive inclusion and exclusion criteria which might have caused limitations in some of the data presented.

In order to obtain a better understanding of the racial/ethnic coverage gaps that are present, with regard to the initiation and completion of the HPV vaccination series, in some cases, further research is needed. A qualitative study designed to examine cultural views and beliefs might shed light on some of the factors contributing to the unequal distribution of coverage. This proposed study should examine the various ethnic/racial groups found in the United States across different regions of the country to examine the varying beliefs that might present barriers to the initiation and completion of the vaccination series.

## 4. Results

### 4.1. SEER Data

**Figure 1:**
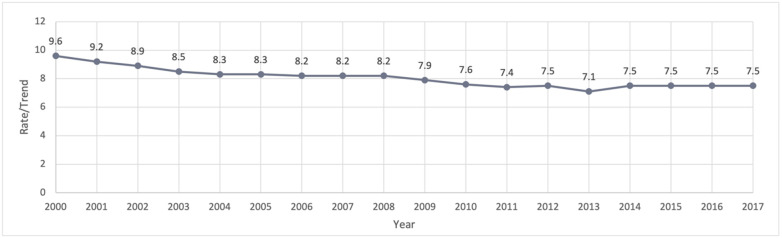
Incidence rate of malignant cervical cancer-SEER Research Data, 18 Registries (excl AK), November 2019 sub (2000–2017).

The incidence rate trend analysis performed on the SEER data for malignant cervical cancer found that the rate of diagnosis has been falling over the nearly two decades examined in this study. In 2000, the incidence rate for malignant cervical cancer diagnoses was 9.6, and by 2007, the rate reduced to 8.2. Since around 2015, the incidence rate for malignant cervical cancer diagnoses leveled out to 7.5, and this rate has remained consistent for the most recently released data, which pertains to the year 2017. There are years of fluctuation, with slight increases and decreases across the seventeen years evaluated, but overall, the rate of incidence is steadily decreasing across the U.S. population, which is illustrated below in Table 1 and Figure 1.

### 4.2. NIS-Teen Data

**Figure 2:**
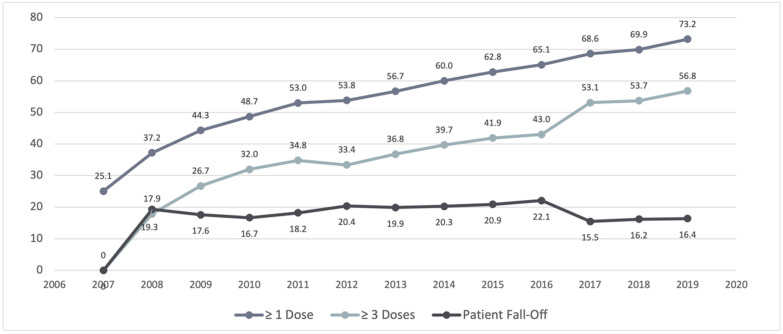
Estimated HPV vaccine coverage (by percentage) among adolescents aged 13–17 by year, dose, and patient fall-off/incompletion-NIS Teen 2007–2019.

The National Immunization Survey’s (NIS) teenage results indicate that since 2007, female vaccination coverage has gradually increased, as illustrated below in Figure 2. According to their records, in 2007, an estimated 25.1% of females within the ages of 13–17 had initiated the HPV vaccination series, thus demonstrating that ≥1 dose had been administered at the time the survey was conducted. By 2010, the coverage for females increased to an estimated 48.7% for those who received ≥1 dose, and 32.0% for those who had received ≥3 doses at the time the survey was conducted. The HPV vaccine coverage continued to increase steadily each year with little disruption, and in the most recent documented results (2019), 73.2% of females aged 13–17 had initiated vaccination (≥1 dose), and 56.8% had completed the vaccination series. Analysis also revealed that there was a substantial widening of the gap between initiation and completion between the years 2012–2016, before a large uptick towards completion in 2017, illustrated clearly in Figure 1.

## 5. Discussion

The incidence rate of malignant cervical cancer has fallen significantly; however, it is still a present danger to the health of females not only in the United States, but globally. The fact that we have an available method that can prevent significant levels of disease and deaths associated with dangerous strains of the human papillomavirus but are failing to protect our population more effectively through its utilization, is concerning. We know that the HPV vaccine can effectively protect against the most common causal factors associated with cervical cancer, but we still have not even been able to meet the desired initiation rates. Greater levels of focus and energy dedicated to increasing our initiation and completion rates of the HPV vaccination series need to be further established. Those engaged in planning this need to be conscious of the unequal distribution of knowledge that has already been detected across race/ethnicity and socioeconomic status, in the hope of reducing the present gaps that have been detected in the population.

In Figure 2, the illustration clearly depicts that between the years 2012–2016, there was a widening of the gap between adolescent females that initiated (≥1 dose) the vaccination series, and those who completed the vaccination series (≥3 dose). The issue of failing to complete the full three-dose vaccination series is present in every year, but this specific time period showed increased rates of failure. These years occur after the establishment of the Affordable Care Act, so the price of vaccination should not have played a role since the HPV vaccine would fall under the category of preventative care, and therefore, the costs would have been entirely covered by the patient’s choice of insurer. It is also unlikely that a lack of insurance would have explained this gap, since the rate of uninsured people in the U.S. population decreased during these years. Further research into these specific years would need to occur in order to better explain this occurrence. Additionally, companies that make HPV vaccines can look into whether one dose would be strong enough to prevent future infection, or if three doses are necessary. Evidence of a single dose of the COVID-19 vaccine being effective against hospitalization and death has been established in the literature [20] (pp. 2187–2201). This lends support to developing one dose of the HPV vaccine, so that patients who are supposed to take three doses do not fall through on their vaccination schedule after the first dose.

Raising the awareness of the dangers associated with HPV needs to be a focus of public health officials at federal, state, and local levels. The language used when communicating these known and scientifically proven dangers needs to be carefully chosen and should be written at a sixth grade reading level to improve the odds of overall comprehension. The messaging needs to be culturally conscious and inclusive to better register with the overall U.S. population which is very diverse. Specialized marketing (brochures, fact sheets, and informational videos) paraphernalia should be designed to target specific audiences found in the subgroups of our country, based on age, language, and religious and cultural views. Each of these subgroups is more likely to engage with messaging if the message is delivered in a way that is understandable and relatable. Communication is a tremendously important factor in the decision to initiate and complete a vaccination series, and with that in mind, we need to produce content that, at the very least, encourages the audience to begin a conversation with their healthcare provider.

There are specific initiatives that are needed to expand HPV vaccine coverage, reduce the risk of cervical cancer, and reduce cervical cancer rates. According to the recommendation by the World Health Organization, the HPV vaccine should be a part of the national immunization program [21] (pp. 102–106). Another strategy would be to vaccinate girls aged from 9–14, as this can be incorporated into a public health program for school age girls. Service platforms for this vaccination program could be public schools, private schools, and community colleges. Health service organizations that partner with the community, and a strong communication and social movement are imperative [21] to reduce operational costs related to this vaccination, and to expand the reach of this vaccine.

Healthcare providers need to be given access to information and training on the best methods of communication when educating and recommending vaccinations to a diverse patient pool. Cultural competency is key for all medical practice, but with the growing number of conspiracies, and the sensitivity that surrounds vaccines in the current climate, using appropriate communication methods has become even more essential. Possessing knowledge of the safety and precautionary measures that are involved in both the manufacturing and administration of the HPV vaccine will be helpful, but skills in de-escalation and sensitive communication are also becoming increasingly valuable. The support and encouragement of providers from the view of the patient is consequential in some cases, and thus, instilling confidence and garnering the support of healthcare providers is key. Primary care physicians have proven to be supportive of a harmonized approach to HPV vaccination recommendations across all genders, and in doing this, they could avoid confusion and ensure that all eligible patients are equally informed [22] (p. 3699). By harmonizing the recommendation and removing the concern of gender and only tracking age, physicians would be able to streamline their vaccination recommendations with greater ease. Inheriting this practice could prove to be beneficial for providers that already have to deal with heavy caseloads, and therefore, it is highly recommended.

Healthcare administrators need to work in conjunction with public health officials and organizations, as well as clinical staff, to create procedures designed to help encourage vaccination utilization. It is the responsibility of the administration to ensure that the education, equipment, and support needed by these professionals are made available, and that these professionals feel supported and encouraged to communicate any needs deemed necessary to support their efforts in increasing the rates of vaccination. Administrators need to be aware of the vaccination utilization rates in their community, and they need to be tracking any changes that are communicated from manufacturers to ensure that quality and supplies remain consistent within their organization. It is important that the administrator also monitors the organizational culture surrounding vaccination and patient communication. Internal communication will assist in determining where areas of improvement are needed within the organization, with regard to organizational culture. If the goal is to increase the rates of vaccination in patients associated with the organization, it will be important to ensure that everyone within the organization is aware and is in support of the overall effort. If people are not supportive and engaged in the efforts, it is unlikely that we will reach the American Cancer Association HPV vaccination initiation goal in 2026 (80% initiation rate). The level of HPV vaccination recommendations that are provided by physicians, and other clinical staff, to eligible candidates should be tracked and benchmarked. Along with organization-wide vaccination initiation and completion goals, expectations should be clearly set and internally displayed. With clearly set goals, members of the organization are more likely to feel that efforts are being invested in universally, and therefore, this will hopefully enhance motivation.

Due to the COVID-19 pandemic, that broadly took hold in early 2020, it is highly likely that we will see disruption in the initiation and completion of HPV vaccinations that were planned or continued into the year 2020. In an attempt to contain the spread of the virus, states across the United States entered mandatory lockdowns and business closures to varying degrees, but these policies resulted in the halt of all non-essential medical practice as well. Once many of these policies were reduced or withdrawn, and when businesses began to reopen, it was found that anxieties and fears of infection were still affecting people’s behaviors. Many continue to avoid unnecessary trips outside of the home, and some may even be avoiding medical facilities due to fear of possible infection. This change in behavior is likely to be represented in the rate of HPV vaccine utilization (initiation and completion) for the year 2020, but we will have to wait for the results to be shared to truly see the impact. This is concerning because those who had previously initiated vaccination prior to, or going into early 2020, will likely experience disruption to their vaccination schedule. Disruption of the vaccination schedule is concerning in terms of whether it will cause weakened effectiveness, which could mean these individuals’ will not receive the same protective benefits as those who were able to complete their series in accordance with the designated schedule. It will likely take years for us to see the ramifications of this disruption, and a push for ‘catch-up dosing’ will need to be encouraged in the hope of providing the best protection possible given the circumstances.

## 6. Conclusions

The incidence rate of cervical cancer has been falling across the United States, but at a rate that now appears to have plateaued. The HPV vaccine, when administered according to the manufacturer’s guidelines, appears to offer effective protection against the most hazardous types of HPV, which are known to be causal factors in the majority of malignant cervical cancer cases. Further support, and a push from physicians and public health officials, will likely be necessary for the rate of HPV vaccine utilization to increase over the coming years. A collaborative effort between public health officials, medical professionals, and healthcare administrators is needed if we hope to reach the 2026 HPV Cancer Free goals set out by the American Cancer Society.

In the United States, further research is needed to better understand the disproportionate rate at which HPV vaccination series are not being completed among racial/ethnic minorities, compared with the completion rates of White people. Research has determined that these are statistically significant rates, with Black, Latina, and Asian women completing the series at lower rates than those seen in White women. Socioeconomic status has already been determined as a factor when measuring for HPV and HPV vaccine knowledge, but research into how cultural factors can contribute to these results would prove to be useful. With a better understanding, the professionals involved can adjust their approach accordingly, with the hope of increasing the rate of vaccination series completion as a result.

## Figures and Tables

**Table 1:** Estimated HPV vaccine coverage among adolescents aged 13–17 by year, dose, and patient fall-off/incompletion–NIS Teen 2007–2019 (shown in percentages).

Doses	2007	2008	2009	2010	2011	2012	2013	2014	2015	2016	2017	2018	2019
≥1 dose	25.1	37.2	44.3	48.7	53.0	53.8	56.7	60.0	62.8	65.1	68.6	69.9	73.2
≥3 doses		17.9	26.7	32.0	34.8	33.4	36.8	39.7	41.9	43.0	53.1	53.7	56.8
Patient Fall-off		19.3	17.6	16.7	18.2	20.4	19.9	20.3	20.9	22.1	15.5	16.2	16.4

## Data Availability

Data can be availed at https://healthcaredelivery.cancer.gov/seermedicare, accessed on 23 March 2022.
